# Establishment of Human Neural Progenitor Cells from Human Induced Pluripotent Stem Cells with Diverse Tissue Origins

**DOI:** 10.1155/2016/7235757

**Published:** 2016-04-26

**Authors:** Hayato Fukusumi, Tomoko Shofuda, Yohei Bamba, Atsuyo Yamamoto, Daisuke Kanematsu, Yukako Handa, Keisuke Okita, Masaya Nakamura, Shinya Yamanaka, Hideyuki Okano, Yonehiro Kanemura

**Affiliations:** ^1^Division of Regenerative Medicine, Institute for Clinical Research, Osaka National Hospital, National Hospital Organization, Osaka 540-0006, Japan; ^2^Division of Stem Cell Research, Institute for Clinical Research, Osaka National Hospital, National Hospital Organization, Osaka 540-0006, Japan; ^3^Department of Physiology, Keio University School of Medicine, Tokyo 160-8582, Japan; ^4^Center for iPS Cell Research and Application (CiRA), Kyoto University, Kyoto 606-8507, Japan; ^5^Department of Orthopedic Surgery, Keio University School of Medicine, Tokyo 160-8582, Japan; ^6^Department of Neurosurgery, Osaka National Hospital, National Hospital Organization, Osaka 540-0006, Japan

## Abstract

Human neural progenitor cells (hNPCs) have previously been generated from limited numbers of human induced pluripotent stem cell (hiPSC) clones. Here, 21 hiPSC clones derived from human dermal fibroblasts, cord blood cells, and peripheral blood mononuclear cells were differentiated using two neural induction methods, an embryoid body (EB) formation-based method and an EB formation method using dual SMAD inhibitors (dSMADi). Our results showed that expandable hNPCs could be generated from hiPSC clones with diverse somatic tissue origins. The established hNPCs exhibited a mid/hindbrain-type neural identity and uniform expression of neural progenitor genes.

## 1. Introduction

Human neural progenitor cells (hNPCs), which are present in fetal and adult neural tissues, have the potential to be therapeutically beneficial in the treatment of neuronal diseases such as spinal cord injury or stroke; however, it is technically difficult to obtain hNPCs from human neural tissues. The development of human embryonic stem cells (hESCs) [1] and human induced pluripotent stem cells (hiPSCs) [2, 3] has greatly improved the prospects of regenerative medicine. We are now able to obtain unlimited hiPSCs from every somatic tissue source [4]. However, hiPSC clones exhibit variable differentiation propensities [5], similar to hESCs [6].

Many protocols have been reported for the neural induction of hESCs/hiPSCs. Transplantable neural precursors were first derived from hESCs, which were subjected to spontaneous embryoid body (EB) formation, followed by neural rosette selection [7]. EB-mediated neural rosette formation is used not only for establishing rosette-stage neural stem cells (R-NSCs) from hESCs [8], but also for generating long-term self-renewing neuroepithelial-like stem (lt-NES) cells from hESCs/hiPSCs [9, 10]. However, the neural induction efficiency of these methods depends on the innate differentiation propensity of the hESC/hiPSC clones [11]. Using a strategy based on the neural default model, inhibitors of the bone morphogenic protein (BMP) signaling pathway, such as Noggin or the small molecule Dorsomorphin, have been used to direct the differentiation of hESCs/hiPSCs toward the neural lineage [12]. In addition, Lefty-A or the small molecule SB431542 can be used to inhibit Nodal, a member of the transforming growth factor (TGF) *β* family which contributes to endodermal or mesodermal fate selection, promoting neural induction of hESCs. The combination of a BMP antagonist and a TGF*β*/Activin/Nodal inhibitor has been used to accelerate the neural induction of hESCs/hiPSCs [11, 13–15]. To establish a reproducible EB-based method, Eiraku et al. subjected fully dissociated hESCs/hiPSCs to SFEBq (serum-free culture of EB-like aggregates) in the presence of a Rho-associated protein kinase (ROCK) inhibitor, resulting in the formation of uniformly sized EBs [16]. It has also been shown that neural precursor cells can be derived from hESCs at physiological oxygen levels (3 to 5%) [17].

Neural induction using these methods has been successful for only a limited number of hESC/hiPSC clones. Koyanagi-Aoi et al. recently reported on the SFEBq-mediated induction of dopaminergic neurons from hESCs and hiPSCs derived from various somatic tissues [18]. The aim of the current study was to determine whether hNPCs can be derived from any hiPSC clone regardless of its somatic tissue origin. We evaluated 21 hiPSC clones derived from human dermal fibroblasts (HDFs, 13 clones), cord blood (CB) cells (3 clones), and peripheral blood mononuclear (PBMN) cells (5 clones) using an EB formation-based method (EBFM) and an EB formation method that includes dual SMAD inhibitors (dSMADi). Although there is consensus that SMAD inhibition is necessary for neural induction as mentioned above, there are many variations among methods. Therefore, we performed the dSMADi method, in which the conditions are easily controlled, using two types of media and two different oxygen levels. Thus, the previously reported neural induction method was expanded into four conditions. Our data indicate that dual SMAD inhibition can be used to generate mid/hindbrain-type hNPCs from hiPSCs regardless of their somatic tissue origin. These expandable hNPCs may be a useful cell source for regenerative medicine research and the treatment of neuronal diseases.

## 2. Materials and Methods

### 2.1. Culture of hiPSCs

This study was conducted in accordance with the principles of the Helsinki Declaration, and the use of hiPSC clones was approved by the ethics committee of Osaka National Hospital (number 110) and CiRA, Kyoto University. All hiPSC clones (Table S1 in Supplementary Material available online at http://dx.doi.org/10.1155/2016/7235757) were cultured at the CiRA on mitomycin C-treated SNL feeder cells in primate ES cell medium (ReproCELL) until ~50% confluent and then transported to our laboratory at Osaka National Hospital. The hiPSC clones were cultured for two days before neural induction.

### 2.2. Neural Induction of hiPSCs

Two neural induction methods were used in this study: EBFM [19, 20] and an EB formation method using dual SMAD inhibitors (dSMADi) [14, 16]. Each of the hiPSC clones was simultaneously subjected to neural induction using the two methods.

For EBFM, the hiPSCs were treated with 10 *μ*M Y-27632 (ROCK inhibitor) for 1 h at 37°C and then detached using 1 mg/mL collagenase IV (Life Technologies) and floated onto gelatin-coated dishes to remove the SNL feeder cells. After 30 min, floating EBs were transferred to Petri dishes containing DMEM/F12 (D6421, Sigma) with 20% knockout serum replacement (KSR, Life Technologies), 0.1 mM nonessential amino acids (NEAA, Life Technologies), 2 mM L-glutamine (Life Technologies), 0.1 mM 2-Mercaptoethanol (2-ME, Life Technologies), antibiotic-antimycotic (Life Technologies), and 10 *μ*M Y-27632 (day 0). The next day (day 1), the medium was replaced with 5% KSR-containing medium and cultured further for 30 days with a medium change every two days.

For dSMADi, hiPSCs were treated with 10 *μ*M Y-27632 for 1 h at 37°C and then dissociated with Trypsin/EDTA to generate single-cell suspensions and suspended in two types of medium: KSR-based medium [DMEM/F12 (D6421) with 20% KSR, 0.1 mM 2-ME, 10 *μ*M SB-431542 (SB, Sigma), and 2 *μ*M Dorsomorphin (DSM, Wako)] and B27N2-based medium [DMEM/F12 (D8062) with 15 mM HEPES, 5% B27, 5% N2 supplement (N2, Life Technologies), 10 *μ*M SB, 2 *μ*M DSM, and 10 ng/mL bFGF]. Both media were supplemented with 30 *μ*M Y-27632 for the first 3 days. Completely dissociated cells were then seeded into ultralow attachment 96-well plates (PrimeSurface® 96-well, Sumitomo Bakelite) at 9,000 cells/well, centrifuged at 700 rpm for 3 min (quick-aggregation), and cultured in a 5% CO_2_ incubator with 5 or 20% O_2_. Thus, the dSMADi neural induction was conducted using four conditions: KSR/20% O_2_, KSR/5% O_2_, B27N2/20% O_2_, and B27N2/5% O_2_. The cells were cultured for 14 days with daily replacement of half the spent medium with fresh medium. On day 14, the aggregates were dissociated mechanically and cultured on Petri dishes in a 5% CO_2_ incubator with 20% O_2_ to generate the first passage of hNPCs. Neurospheres were generated from the second passage of NPCs by completely dissociating the cells with Accutase*™* (Innovative Cell Technologies) and then cultured on nontreated flasks. If the hNPCs attached to the culture vessels at early passages, we used ultralow attachment dishes (PrimeSurface, Sumitomo Bakelite) to establish the hNPCs as neurospheres.

### 2.3. Maintenance of hNPCs

The hNPCs were seeded at 1 × 10^5^ cells/mL and cultured as floating neurospheres in hNPC medium [DMEM/F12 (D8062) with 15 mM HEPES, 2% B27, 20 ng/mL EGF (PeproTech), 20 ng/mL FGF2 (PeproTech), 10 ng/mL leukemia inhibitory factor (Millipore), and 5 *μ*g/mL heparin (Sigma-Aldrich)].

### 2.4. Quantitative Reverse Transcription-Polymerase Chain Reaction (Quantitative RT-PCR)

Total RNA was extracted using the RNeasy MinElute Cleaning Kit (Qiagen), and the cDNAs were synthesized using the PrimeScript® RT Master Mix (Takara Bio) according to the manufacturer's specifications. Quantitative PCR analysis was performed using gene-specific primers (Table S4), the Power SYBR® Green PCR Master Mix, and the 7300 Real-Time PCR System (Applied Biosystems). Gene expression levels were expressed as delta Ct values normalized to* GAPDH* [23].

### 2.5. Measurement of Neural Aggregate Size

Phase-contrast images of eight wells per condition for each clone were captured (one representative image per condition is shown in Figure S3). The projected areas of the neural aggregates were measured using ImageJ [24]. The aggregate size was calculated as a sphere volume using the circular diameter determined from the projected area [25].

### 2.6. In Vitro Neuronal Differentiation

To avoid disturbing the naturally formed niche, the neurospheres were not dissociated. The intact neurospheres were transferred to vessels coated with Growth Factor Reduced Matrigel*™* (diluted to 1 : 30, BD Biosciences) and cultured in Neurobasal Medium (Life Technologies) containing 2% B27 and 1% L-glutamine for 2 weeks [22].

### 2.7. Immunocytochemical Staining

Cells were fixed in 4% paraformaldehyde and washed with PBS. The fixed samples were then blocked with 10% normal goat serum and incubated with anti-*β*III tubulin antibody (clone TuJ1, Babco) overnight at 4°C. The samples were then incubated with AlexaFluor-488-conjugated goat anti-mouse IgG (Molecular Probes, Life Technologies) for 1 h at room temperature. The stained samples were examined with a confocal laser-scanning microscope. All staining procedures were performed with matched-isotype controls [22].

### 2.8. Neurite Analysis

*β*III tubulin-positive neurites in four regions were detected by appropriate thresholding and then skeletonized using ImageJ [24]. Total neurite length was determined by counting the positive pixels [26].

### 2.9. Statistical Analysis

Significant differences in gene expression levels obtained by quantitative RT-PCR were analyzed using the Steel-Dwass nonparametric multiple comparison test or Welch's *t*-test. Significant differences in neural aggregate size were also analyzed by the Steel-Dwass comparison test. Significant differences in total neurite length were analyzed by Dunnett's test. See figure legends for details.

## 3. Results and Discussion

**Figure 1:**
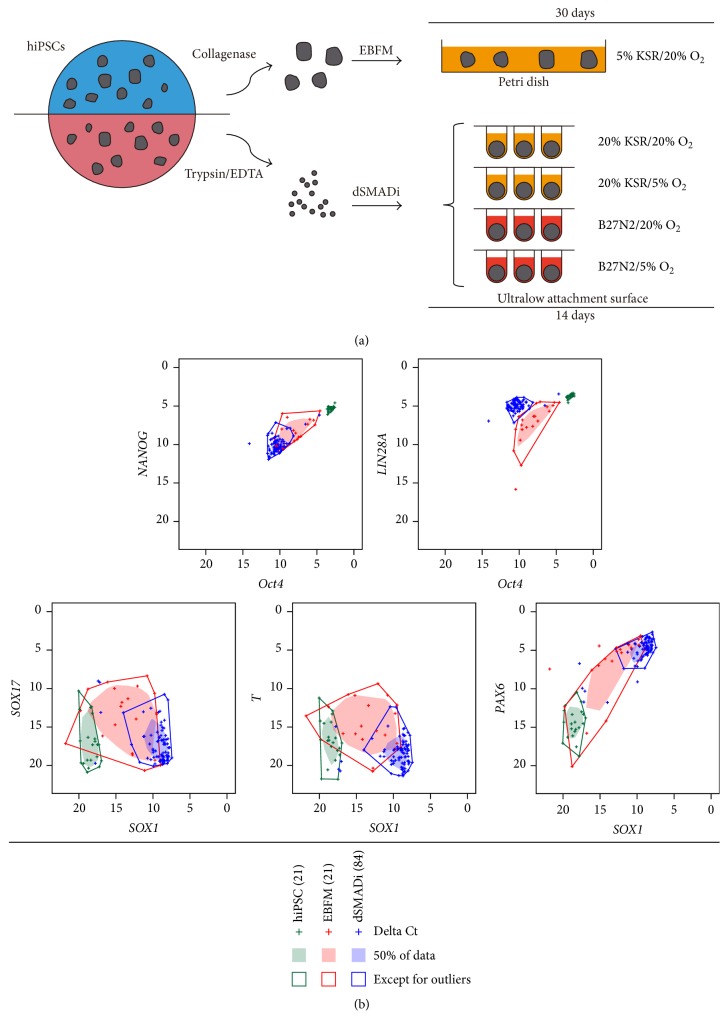
dSMADi improves the neural induction efficiency of hiPSCs regardless of their somatic tissue origin. (a) Schematic drawing of the neural induction methods used in this study. (b) Bivariate box plots displaying gene expression levels in hiPSCs, day 30 EBFM-derived EBs, and day 14 dSMADi-derived aggregates. Quantitative RT-PCR-generated delta Ct values for the pluripotency marker genes (*Oct4*,* NANOG,* and* LIN28A*), endoderm marker gene (*SOX17*), mesoderm marker gene (*T*), and neural marker genes (*SOX1* and* PAX6*) are shown. Green: hiPSCs; red: EBFM; blue: dSMADi. The numbers of clones analyzed are indicated in parentheses and “+” symbols represent each of the delta Ct values. Filled-in regions contain the 50% of the data points, and data points outside of the surrounding lines represent outliers. See also Figures S1 and S2.

There are many variations among neural induction methods, although there is consensus about the necessity of SMAD inhibition. To determine whether hiPSC clones derived from different somatic tissues could differentiate into hNPCs without specific neural induction methods (Figure 1(a)), we examined 21 hiPSC clones established in CiRA (Table S1). These hiPSC clones were derived from three different tissues: HDFs, CB cells, and PBMN cells (Table S1).

All of the hiPSCs exhibited a typical undifferentiated hESC-like morphology (Figure S1A). Quantitative RT-PCR showed that the clones expressed uniformly high levels of the pluripotency marker genes* Oct4*,* NANOG*, and* LIN28A* but very low levels of the differentiation marker genes* SOX17* (endoderm),* T* (mesoderm), and* SOX1* and* PAX6* (both neural) just prior to neural induction (Figure 1(b) and Figure S1B).

Two predominant methods for inducing the neural differentiation of hESCs/hiPSCs are the EB formation-based method (EBFM) and EB formation with dual SMAD inhibitors (dSMADi). We controlled the aggregate size in the dSMADi method using a quick-aggregation procedure [16] and examined four additional conditions using this method by assessing combinations of two culture media and two different oxygen levels. In the EBFM approach, we used a low concentration (5%) of knockout serum replacement (KSR) [19, 20] to limit the amount of BMP-like activity [27], which opposes neural induction and is present in the KSR.

We subjected the 21 hiPSC clones to the five different neural induction procedures (Figure 1(a)). To assess the neural induction efficiency using dSMADi, we compared gene expression levels among the hiPSCs, day 30 EBFM-derived EBs, and day 14 dSMADi-derived aggregates, by quantitative RT-PCR (Figure 1(b), Figure S1B, Figure S1C, and Figure S2). Bivariate box plots showed that the pluripotency marker genes,* Oct4* and* NANOG,* were strongly and uniformly downregulated in day 14 dSMADi-derived aggregates but not in day 30 EBFM-derived EBs, which exhibited more variable expression among the clones (Figure 1(b) and Figure S1C). Interestingly, dSMADi treatment also resulted in the slight downregulation of another pluripotency marker gene,* LIN28A* (Figure 1(b)).

Day 30 EBs did not uniformly express the endoderm marker gene* SOX17*, mesoderm marker gene* T*, or neural marker genes* SOX1* and* PAX6* and were classified as nonneural, neural, or three germ layer-containing EBs (Figure S1D). In contrast, almost all of the day 14 dSMADi-derived aggregates exhibited the upregulation of both neural marker genes but not nonneural marker genes (Figure 1(b)). These findings indicated that while EBFM-derived EBs exhibited cell lineage variability (only 6 clones differentiated toward specifically neural lineage), dSMADi-derived aggregates exhibited less clonal variation, and almost all of them underwent neural lineage induction regardless of somatic tissue origin and the differentiation protocols (over 90% of clones differentiated toward neural lineage). Hereafter, we will refer to day 14 dSMADi aggregates as neural aggregates.

**Figure 2:**
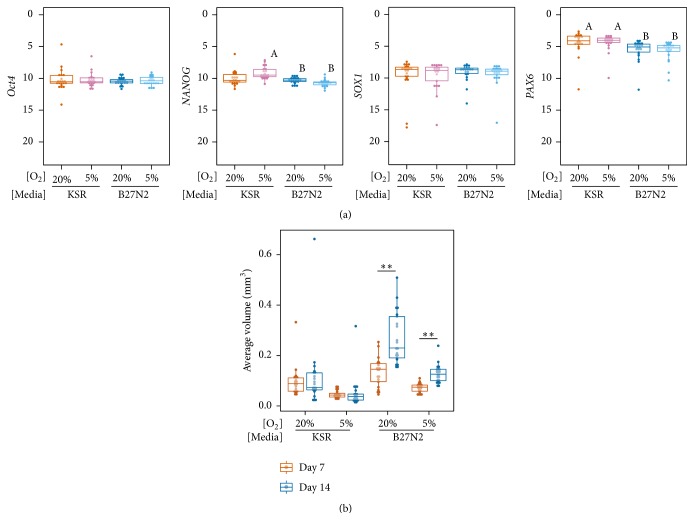
Effects of media and oxygen level on neural aggregate gene expression and size. (a) Dot plots and box plots showing for the gene expression levels of pluripotency markers (*Oct4* and* NANOG*) and neural markers (*SOX1* and* PAX6*) in day 14 aggregates cultured in the various conditions. Twenty-one clones were analyzed in each condition. Statistical significance was determined by the Steel-Dwass test. Statistically significant differences (*p* < 0.01) were found between samples “A” and “B”. See also Figure S2. (b) Dot plot and box plot showing the size of day 7 and day 14 neural aggregates. Statistical analysis was performed with the Steel-Dwass test (^*∗∗*^ *p* < 0.01). All *p* values are shown in Table  S2.

We further evaluated the effects of the four dSMADi conditions by comparing the gene expression levels of the neural aggregates derived in two types of media (KSR or B27N2) and two different oxygen levels (20% or 5%) (Figure 2(a)). Although the expression levels of the pluripotency marker gene* Oct4* and neural marker gene* SOX1* were similar in aggregates cultured in the four conditions, those of* NANOG* in aggregates cultured in KSR/5% O_2_ and of* PAX6* in aggregates cultured in KSR/20% O_2_ and KSR/5% O_2_ were significantly higher than in the B27N2-based conditions (Figure 2(a)). The oxygen level alone did not significantly impact the expression levels of these genes. Although we used a quick-aggregation procedure in the dSMADi experiments to eliminate the size and shape variability observed in EBFM-derived EBs (Figure S1A), we noticed obvious differences in neural aggregate size among the four conditions (Figure S3).

To examine the effects of the culture conditions in detail, we measured the size of the neural aggregates on day 7 and day 14 (Figure 2(b)). The day 14 neural aggregates cultured in B27N2-based conditions were significantly larger than those cultured in KSR-based conditions at each oxygen level, and they were also significantly larger when cultured in 20% O_2_ than in 5% O_2_, in both types of media (Figure 2(b) and Table S2). Notably, neural aggregates did not grow in the KSR-based conditions over the induction period regardless of the oxygen level. In contrast, B27N2, which contains basic fibroblast growth factor, supported the growth of the neural aggregates (Figure 2(b)). These findings indicated that although the four dSMADi conditions efficiently promoted neural lineage induction, the culture medium influenced both gene expression and aggregate size, whereas the oxygen level primarily affected aggregate size.

**Figure 3:**
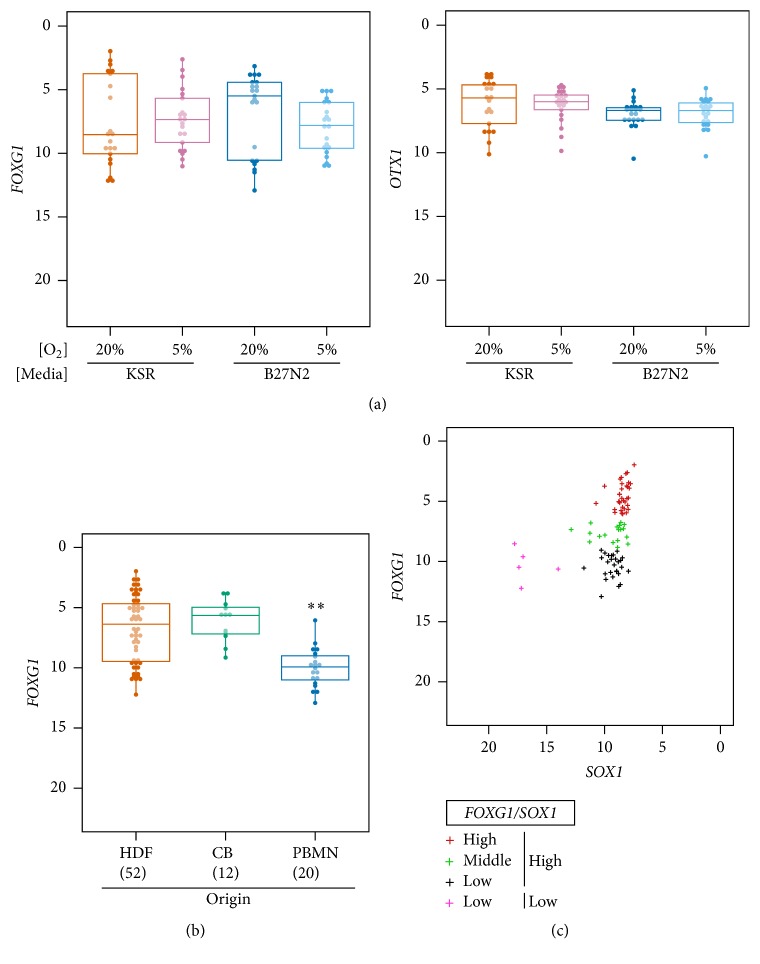
PBMN-derived neural aggregates exhibit low* FOXG1* expression levels. (a) Dot plots and box plots showing* FOXG1* and* OTX1* expression in day 14 neural aggregates cultured in the various conditions. (b) Dot plots and box plots showing the* FOXG1* expression levels according to tissue origin. The number of clones analyzed is indicated in parentheses. Statistical analysis was performed by the Steel-Dwass test.  ^*∗∗*^ *p* < 0.01. (c) Clustering of clones, based on the expression of* FOXG1* and* SOX1*, using the *K*-medoids method. The delta Ct values are indicated by “+” symbols. Four clusters were identified:* FOXG1*-high/*SOX1*-high (red),* FOXG1*-middle/*SOX1*-high (green),* FOXG1*-low/*SOX1*-high (black), and* FOXG1*-low/*SOX1*-low (pink). See also Table S3.

Next, we investigated whether the neural aggregates exhibited a forebrain-type property, as previously observed for lt-NES cells and R-NSCs [8–10]. Because the expression of* PAX6* was higher in KSR-derived aggregates than in B27N2-derived aggregates, we first compared the expression levels of the forebrain marker genes,* FOXG1* and* OTX1*, in day 14 neural aggregates cultured in all four conditions. Although there were no significant differences in* FOXG1* and* OTX1* expression levels among aggregates cultured in the different conditions (Figure 3(a)), the* FOXG1* expression was more variable among the clones compared to the* OTX1* expression. Therefore, we compared the* FOXG1* expression among aggregates derived from somatic tissues of different origins (Figure 3(b)). Notably, the* FOXG1* expression was significantly lower in the clones derived from PBMN cells than in those derived from HDFs and CB cells (Figure 3(b)), although the HDF- and CB cell-derived clones exhibited variable* FOXG1* expression. Given that ES cells generate anterior forebrain-like neural precursor cells in the absence of external signals [28], the variability of* FOXG1* expression in the HDF- and CB-derived clones might reflect variable Wnt activation in the hiPSCs [29].

However, the mechanisms regulating differential* FOXG1* expression between CB- and PBMN-derived clones are unclear because both hiPSC clones were derived from mesodermal tissues, cultured for over 20 passages, and were considered to have lost their somatic tissue-specific epigenetic memory [30–32]. Given that all of the PBMN cells were *αβ*T cells in this study, their genomes were modified due to T-cell receptor (TCR) rearrangement, in contrast to the HDF and CB cells. The genes encoding T-cell receptor alpha (*TRA*) and T-cell receptor beta (*TRB*) are located at chromosomal regions 14q11.2 and 7q34, respectively.* FOXG1* is located at chromosomal region 14q13. Although TRA and* FOXG1* are separated by about 6 million bases, some epigenetic modifications might still occur in the regulatory region of* FOXG1*.* FOXG1* is critical for normal corticogenesis [33]; therefore, PBMN-derived hiPSCs might not be a good source for analyzing normal cortical development or disease modeling. Further studies are required to elucidate whether inhibition of Wnt signaling improves the induction of anterior neural progenitors from PBMN-derived clones. On the other hand, we hypothesized that neural aggregates with the potential for producing uniform hNPCs could be identified by screening for* FOXG1* expression. To evaluate this possibility, we grouped all of the neural aggregates into four categories (*FOXG1*-high/*SOX1*-high,* FOXG1*-middle/*SOX1*-high,* FOXG1*-low/*SOX1*-high, and* FOXG1*-low/*SOX1*-low) by clustering based on* FOXG1* and* SOX1* expression levels (Figure 3(c) and Table S3). We then selected neural aggregates from each category, except for the* SOX1*-low, nonneural lineage category, for further expansion. Notably, these neural aggregates included clones derived from each of the three different tissue types (Table S3).

**Figure 4:**
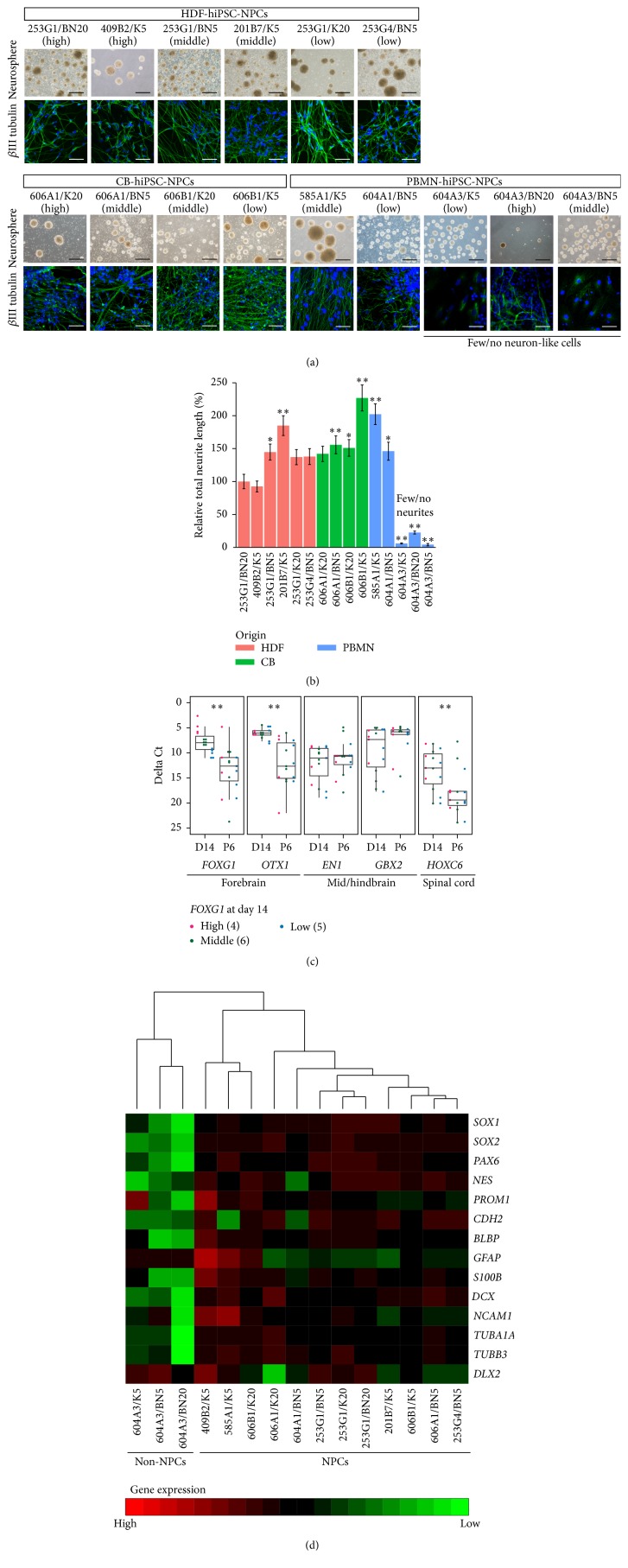
Established hNPCs express regional markers indicative of a mid/hindbrain-type property. (a) Morphologies of the expanded neurospheres at approximately passage 6 (upper panel). *β*III tubulin-positive neurons after 14 days of neuronal differentiation. Green: *β*III tubulin; blue: nucleus (lower panel). Scale bars: 500 *μ*m (black) and 50 *μ*m (white). (b) Neurite analysis after 14 days of neuronal differentiation. Relative total neurite length is shown as the mean ± SD. Statistical analysis was performed using Dunnett's test.  ^*∗*^ *p* < 0.05 and ^*∗∗*^ *p* < 0.01. (c) Dot plots and box plots showing the expression of forebrain markers (*FOXG1* and* OTX1*), mid/hindbrain markers (*EN1* and* GBX2*), and a spinal cord marker (*HOXC6*) in day 14 (D14) neural aggregates and neurospheres at approximately passage 6 (P6). The numbers of clones analyzed are shown in parentheses. Statistical analysis was performed using Welch's two-sample *t*-test.  ^*∗∗*^ *p* < 0.01. (d) Hierarchical clustering of clones based on neural progenitor marker expression. Red: high gene expression; green: low gene expression.

To facilitate the expansion of homogeneous populations of hNPCs, we cultured them as floating neurospheres [22] rather than as adherent cells like lt-NES cells. Expandable neurospheres were established at approximately passage 6 regardless of the* FOXG1* expression level in day 14 neural aggregates or the somatic tissue origin (Figure 4(a)). To confirm that the neurospheres were hNPCs, neuronal differentiation was induced using the serum-free neuronal differentiation protocol. All of the HDF- and CB cell-derived neurospheres differentiated into *β*III tubulin-positive neurons with long neurites, whereas only two of the five PBMN cell-derived neurospheres underwent neuronal differentiation (Figures 4(a) and 4(b)). Notably, all of the clones that failed to differentiate were derived from the hiPSC clone 604A3 (Figures 4(a) and 4(b)). These results also indicated that hNPC establishment was independent of the* FOXG1* expression level in day 14 neural aggregates and of the hiPSC somatic tissue origin.* FOXG1* expression decreases with increasing passage of lt-NES cells [9, 10] and neurospheres [22]. Thus, we hypothesized that* FOXG1* expression in day 14 neural aggregates may decrease over time, resulting in the formation of mid/hindbrain-type progenitors. To assess this supposition, we compared the expression of regional identity marker genes in day 14 neural aggregates and passage 6 neurospheres (Figure 4(c)). We found that the neural aggregates expressed variable levels of not only the forebrain markers,* FOXG1* and* OTX1*, but also each marker gene for the mid/hindbrain (*EN1*/*GBX2*) and spinal cord (*HOXC6*) (Figure 4(c)). These broad expression patterns of regional marker genes were consistent with a previous report on the activity of endogenous Wnt signaling [34]. In contrast, passage 6 neurospheres expressed higher and less variable levels of the mid/hindbrain markers and reduced levels of the forebrain and spinal cord markers (Figure 4(c)). Thus, while the day 14 neural aggregates exhibited upregulated forebrain marker expression, established hNPCs displayed a mid/hindbrain-like regional property, consistent with previous findings [22].

Finally, we compared the expression of neural progenitor genes among the established hNPCs (Figure 4(d)). We found that hNPCs and 604A3-derived non-hNPCs were separately clustered and that the hNPCs exhibited similar neural progenitor gene expression patterns regardless of the dSMADi conditions and their somatic tissue origin. All of the PBMN clones met our criteria of high* SOX1* and* PAX6* expressions on day 14 (Figure 3(c)). All three of the 604A3 clones had almost the same properties on day 14 but differed from the other clones at passage 6 (Figure 4(d)). We could not eliminate the possibility that the day 14 neural aggregates contained nonneural cells, because gene expression analysis was performed on the bulk population, not at the single-cell level. Therefore, we applied the neurosphere culture method to select a homogeneous population and we were successful in almost every case. However, given that at least neural crest cells can grow in our neurosphere conditions [35], some cell population that was preferentially induced in the 604A3 clone might have been selected and expanded in the neurosphere culture process.

## 4. Conclusions

We conclude that neural lineage cells can be derived from most hiPSC clones, regardless of their somatic tissue origin, using dual SMAD inhibition. We found that PBMN cell-derived hiPSC clones did not exhibit increased expression of the forebrain marker gene,* FOXG1*, but generated hNPCs with neuronal differentiation ability as efficiently as HDF- and CB-derived hiPSC clones. Moreover, neural aggregates at the early neural induction stage exhibited variable neural regional marker gene expression patterns and gave rise to hNPCs that uniformly exhibited a mid/hindbrain-type property and expressed similar levels of neural progenitor genes. These findings suggest that the hNPCs described here may be a useful cell source for basic and pharmaceutical research aimed at developing regenerative therapies for treating various neuronal diseases.

## Supplementary Material

The supplementary material contains three figures (Figure S1, S2, and S3) and four tables (Table S1, S2, S3, and S4). Figures S1, S2, and S3, which are related to Figure 1 and 2, show the morphologies and delta Ct values for gene expression in hiPSCs, EBFM-derived EBs, and dSMADi-derived neural aggregates. Table S1 shows the information regarding the hiPSCs used in this study. Table S2, which is related to Figure 2, compares the effects of two types of culture media and oxygen levels on the size of day 7 and day 14 neural aggregates. Table S3, which is related to Figure 3, shows the list of neural aggregates clustered based on *FOXG1* and *SOX1* expression levels. Table S4 presents the primer sequences used for quantitative RT-PCR.
