# Supplementary material for: Establishment of Human Neural Progenitor Cells from Human Induced Pluripotent Stem Cells with Diverse Tissue Origins
Source: Stem Cells Int. 2016 Apr 26;2016:7235757. doi: 10.1155/2016/7235757 (PMC4861799; doi:10.1155/2016/7235757)
Supplement: Supplementary file 1 — The supplementary material contains three figures (Figure S1, S2, and S3) and four tables (Table S1, S2, S3, and S4). Figures S1, S2, and S3, which are related to Figure 1 and 2, show the morphologies and delta Ct values for gene expression in hiPSCs, EBFM-derived EBs, and dSMADi-derived neural aggregates. Table S1 shows the information regarding the hiPSCs used in this study. Table S2, which is related to Figure 2, compares the effects of two types of culture media and oxygen levels on the size of day 7 and day 14 neural aggregates. Table S3, which is related to Figure 3, shows the list of neural aggregates clustered based on FOXG1 and SOX1 expression levels. Table S4 presents the primer sequences used for quantitative RT-PCR. [file 7235757.f1.pdf]

SUPPLEMENTAL FIGURE LEGENDS

Figure S1, Related to Figure 1.

(A) Morphologies of hiPSCs and EBFM-derived day 9 and day 30 EBs. Scale bar, 500 μm; P, passage number. (B, C) Gene expression panels of hiPSCs (B) and EBFM-derived day 30 EBs (C). The delta Ct values for *Oct4*, *NANOG*, *LIN28A*, *SOX17*, *T*, *SOX1*, and *PAX6* are shown. Red: high expression. (D) Hierarchical clustering of clones in day 30 EBs based on the expression of *SOX1*, *PAX6*, *SOX17*, and *T*.

Figure S2, Related to Figure 1 and Figure 2.

Gene expression panels of the dSMADi-derived day 14 neural aggregates cultured under various conditions. The delta Ct values for *Oct4*, *NANOG*, *LIN28A*, *SOX17*, *T*, *SOX1*, and *PAX6* are shown. Red: high gene expression.

Figure S3, Related to Figure 2.

Morphologies of the day 7 and day 14 neural aggregates generated from the HDF-, CB-, and PBMN-derived clones using dSMADi under various experimental conditions.

Table S1, Related to methods.

Table S2, Related to Figure 2.

Table S3, Related to Figure 3.

Table S4, Related to methods.

Figure S1

A

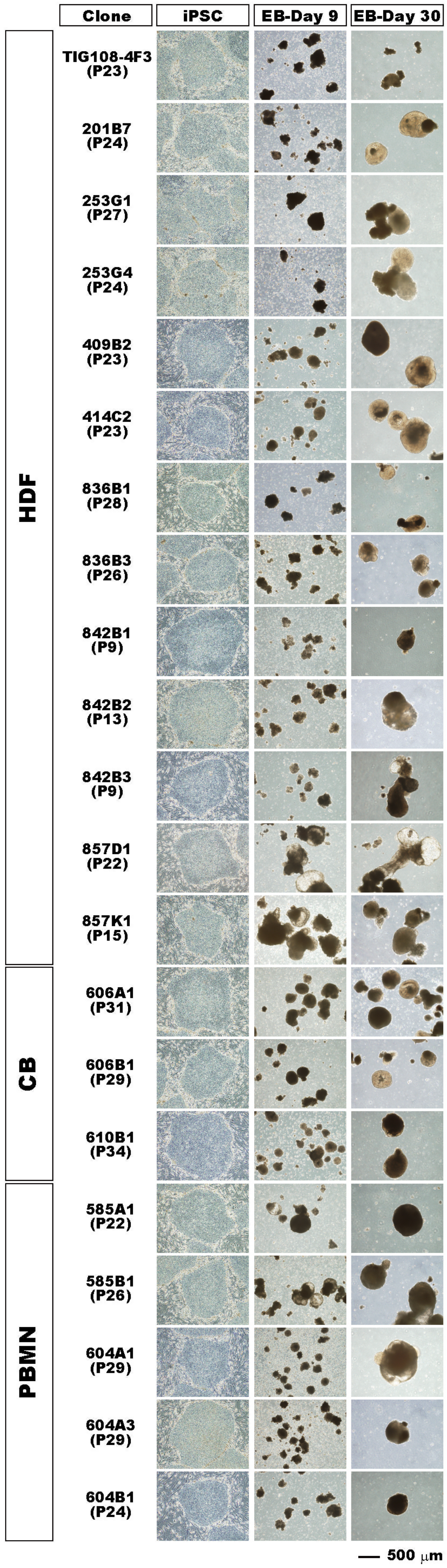

B

iPSC — Delta Ct (scale 0–20)

	Oct4	NANOG	LIN28A	SOX17	T	SOX1	PAX6
TIG108_4F3	2.6	4.6	3.7	19.7	14.5	19.4	16.3
201B7	2.6	5.2	3.4	19.6	21.7	19.7	12.2
253G1	3.4	5.5	3.7	13.2	12.9	18.2	17.5
253G4	3.4	5.1	4.6	20.3	16.7	18.7	16.1
409B2	3.1	5.3	3.8	17.3	17.0	18.1	10.4
414C2	2.7	5.0	3.3	17.3	17.8	17.3	14.5
836B1	3.1	5.2	3.7	20.9	20.6	18.9	15.2
836B3	2.9	5.5	3.4	12.4	13.3	18.3	15.3
842B1	3.0	5.5	3.5	17.3	21.8	17.5	13.0
842B2	3.1	5.3	3.6	20.2	18.6	17.2	13.3
842B3	3.5	5.0	3.7	19.4	17.1	17.0	13.8
857D1	2.8	5.3	3.5	16.9	16.4	17.8	18.8
857K1	3.5	6.1	3.8	10.3	11.2	20.0	17.1
606A1	3.1	5.7	3.5	16.3	16.3	18.8	13.3
606B1	3.0	5.5	3.5	16.5	19.3	17.8	15.1
610B1	3.4	5.3	4.0	18.9	18.1	17.6	11.7
585A1	3.6	5.4	3.8	12.7	14.2	19.9	14.3
585B1	3.4	5.7	4.2	12.5	14.4	18.4	15.6
604A1	3.1	5.3	4.0	19.3	16.8	18.9	14.6
604A3	3.1	5.4	3.8	19.3	17.5	18.4	14.3
604B1	2.8	5.2	3.6	16.8	16.7	19.8	12.8

C

EB-day 30 — Delta Ct (scale 0–20)

	Oct4	NANOG	LIN28A	SOX17	T	SOX1	PAX6
TIG108_4F3	9.7	8.0	12.7	16.2	15.9	16.8	15.8
201B7	7.5	9.0	6.9	13.3	12.1	9.5	3.2
253G1	7.2	9.0	4.5	12.3	14.9	14.3	6.1
253G4	5.9	6.7	5.7	10.0	10.9	15.2	7.0
409B2	9.9	10.3	9.4	19.3	17.8	9.7	4.3
414C2	9.6	10.4	8.1	20.0	17.6	9.0	4.9
836B1	10.7	9.2	10.8	18.6	20.3	12.8	5.1
836B3	10.1	11.0	6.4	20.6	16.0	11.1	4.3
842B1	5.4	6.8	4.9	9.7	9.4	12.1	5.3
842B2	9.7	6.0	7.5	11.8	16.6	14.2	14.2
842B3	4.6	5.6	4.5	11.3	12.2	13.4	6.4
857D1	5.3	7.4	4.5	17.1	13.6	21.8	7.4
857K1	10.5	10.3	15.8	12.9	16.6	19.8	12.4
606A1	9.6	10.6	6.8	13.2	17.1	9.5	3.1
606B1	8.0	9.5	6.3	10.6	13.2	9.8	3.6
610B1	10.5	10.2	8.0	18.4	20.8	12.8	4.7
585A1	8.2	8.2	7.6	10.1	12.4	18.8	20.1
585B1	7.9	8.5	6.3	9.1	10.8	16.1	7.6
604A1	6.2	7.4	5.1	8.3	10.8	10.8	4.7
604A3	7.7	9.3	4.6	14.0	15.5	12.3	4.9
604B1	9.0	6.5	7.8	14.7	15.9	15.1	4.4

D

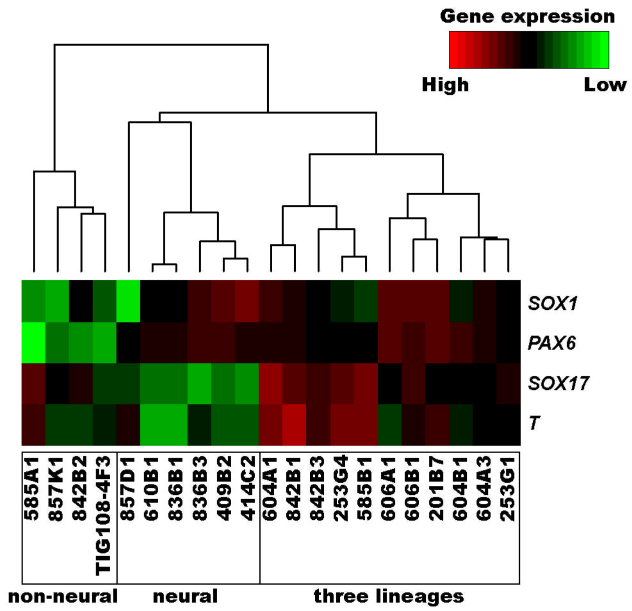

Figure S2

KSR/20% O_2

	Oct4	NANOG	LIN28A	SOX17	T	SOX1	PAX6
TIG108_4F3	4.7	6.2	3.4	19.7	14.9	17.8	6.7
201B7	10.5	10.4	5.0	18.9	19.4	7.9	2.6
253G1	8.1	8.9	4.5	18.1	12.4	9.7	4.5
253G4	11.4	11.7	5.6	19.8	19.3	9.7	4.4
409B2	11.0	9.2	5.3	17.7	16.1	7.8	3.5
414C2	11.3	9.4	5.3	18.6	20.5	8.9	3.5
836B1	10.8	10.3	4.3	19.5	20.8	8.5	4.6
836B3	10.7	9.1	4.4	17.9	19.7	10.0	5.3
842B1	10.7	10.7	4.3	19.0	19.6	7.4	4.6
842B2	10.5	9.0	4.2	17.2	19.8	8.5	4.5
842B3	10.8	10.4	4.2	18.7	18.4	8.2	5.0
857D1	9.5	10.6	5.4	16.1	19.3	10.3	4.2
857K1	14.1	9.9	6.9	9.2	20.7	17.2	11.7
606A1	10.7	10.4	4.8	18.4	17.3	7.9	2.9
606B1	11.4	10.6	5.0	13.1	21.3	9.3	3.2
610B1	10.4	10.9	5.2	16.1	17.2	8.3	3.4
585A1	9.4	9.9	5.1	12.6	21.1	10.3	4.1
585B1	10.5	10.8	6.0	10.7	16.1	8.5	3.8
604A1	9.6	10.3	4.9	16.6	15.2	8.5	3.3
604A3	10.1	11.1	5.3	18.4	17.0	8.6	3.1
604B1	8.7	9.8	5.0	19.2	18.0	8.8	3.4

KSR/5% O_2

	Oct4	NANOG	LIN28A	SOX17	T	SOX1	PAX6
TIG108_4F3	9.1	7.9	4.4	17.3	16.5	12.9	4.8
201B7	11.6	8.6	5.4	17.3	17.9	8.4	3.4
253G1	6.5	7.4	4.9	17.2	12.4	10.4	6.0
253G4	10.7	10.9	5.3	16.8	21.1	8.8	3.9
409B2	8.6	7.9	5.4	17.2	15.7	8.0	3.8
414C2	10.6	7.1	5.0	16.2	17.9	11.2	4.2
836B1	10.6	9.6	4.9	19.2	18.8	8.0	3.7
836B3	10.3	9.4	4.9	15.1	20.3	8.7	4.1
842B1	11.7	8.8	4.5	17.7	18.0	7.9	4.3
842B2	11.1	8.5	4.6	17.2	18.9	8.4	4.0
842B3	10.5	9.5	4.7	15.1	20.7	8.5	4.5
857D1	10.2	8.9	5.7	16.2	18.8	11.3	5.3
857K1	10.7	9.7	7.2	9.0	20.5	17.4	9.9
606A1	10.3	9.6	5.1	18.3	16.8	8.3	3.3
606B1	10.0	9.3	5.7	14.8	18.9	8.9	3.6
610B1	10.5	9.7	5.2	11.5	19.8	8.0	3.5
585A1	9.8	9.7	5.1	13.0	18.2	11.3	4.1
585B1	10.8	9.8	6.3	12.9	17.8	9.1	4.4
604A1	11.1	10.0	5.6	15.7	17.9	9.4	4.0
604A3	9.9	10.1	5.5	16.7	19.7	8.8	3.4
604B1	9.9	10.1	5.4	16.5	19.4	8.0	3.9

B27N2/20% O_2

	Oct4	NANOG	LIN28A	SOX17	T	SOX1	PAX6
TIG108_4F3	9.4	10.2	3.9	19.2	15.2	8.6	4.8
201B7	9.9	10.3	4.5	19.4	16.8	8.7	5.4
253G1	9.5	9.6	4.8	18.9	18.4	8.6	5.5
253G4	10.7	10.3	5.7	19.4	18.1	9.0	4.0
409B2	10.7	10.3	4.6	19.7	19.4	8.1	4.4
414C2	10.6	9.9	4.3	19.7	17.8	8.5	5.1
836B1	10.7	9.6	4.1	19.7	17.3	8.7	4.4
836B3	10.7	9.7	4.6	19.8	21.4	8.7	4.7
842B1	10.4	10.2	4.1	19.3	20.7	8.0	4.8
842B2	10.3	10.5	3.9	19.4	19.0	9.1	4.2
842B3	10.2	10.0	4.2	18.7	15.9	8.7	4.4
857D1	11.2	11.2	5.2	19.6	18.8	11.8	7.4
857K1	11.1	11.1	5.2	13.1	18.0	14.0	11.8
606A1	10.2	10.0	4.1	18.9	17.8	8.1	4.6
606B1	10.4	10.0	4.1	14.1	18.9	8.4	4.8
610B1	9.9	10.3	4.6	18.8	19.6	7.9	5.3
585A1	9.9	10.4	4.6	18.9	19.0	9.3	5.9
585B1	11.7	11.2	5.3	16.9	17.7	9.4	6.1
604A1	11.4	10.8	5.0	18.9	17.9	9.9	6.4
604A3	10.7	10.6	4.5	19.4	20.4	8.4	5.3
604B1	10.4	10.5	5.1	19.2	19.9	10.3	7.1

B27N2/5% O_2

	Oct4	NANOG	LIN28A	SOX17	T	SOX1	PAX6
TIG108_4F3	9.4	10.5	4.2	19.0	14.9	10.7	4.8
201B7	10.7	11.0	4.4	19.3	18.4	8.6	5.7
253G1	9.6	9.4	5.1	18.0	19.5	10.0	5.5
253G4	11.5	11.9	5.2	20.1	18.0	9.9	9.1
409B2	11.6	10.2	4.9	15.4	20.5	8.8	4.9
414C2	10.2	10.6	4.8	19.8	20.8	8.6	5.5
836B1	10.2	10.4	4.4	16.8	19.3	9.2	4.9
836B3	10.7	10.6	4.3	16.4	18.9	9.1	4.4
842B1	9.1	9.9	4.5	17.0	19.6	8.5	4.7
842B2	10.2	10.6	4.1	19.3	18.1	8.1	4.3
842B3	9.5	10.6	4.4	18.8	16.1	8.6	4.5
857D1	10.8	10.9	5.7	18.3	18.9	10.0	7.1
857K1	10.8	11.1	5.7	13.1	19.5	17.0	10.3
606A1	10.4	10.6	4.1	18.8	18.3	8.8	4.6
606B1	10.0	11.0	4.4	16.1	20.2	8.9	4.8
610B1	9.8	10.7	4.5	16.6	17.5	8.2	5.2
585A1	9.8	10.8	4.8	16.4	16.2	9.2	5.7
585B1	11.5	11.5	4.8	14.4	17.9	8.9	5.5
604A1	10.8	11.2	4.7	18.8	17.6	9.5	6.3
604A3	10.6	10.7	4.7	18.7	17.5	8.1	4.9
604B1	10.7	11.3	4.8	19.3	17.8	9.0	7.3

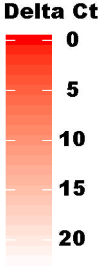

Figure S3

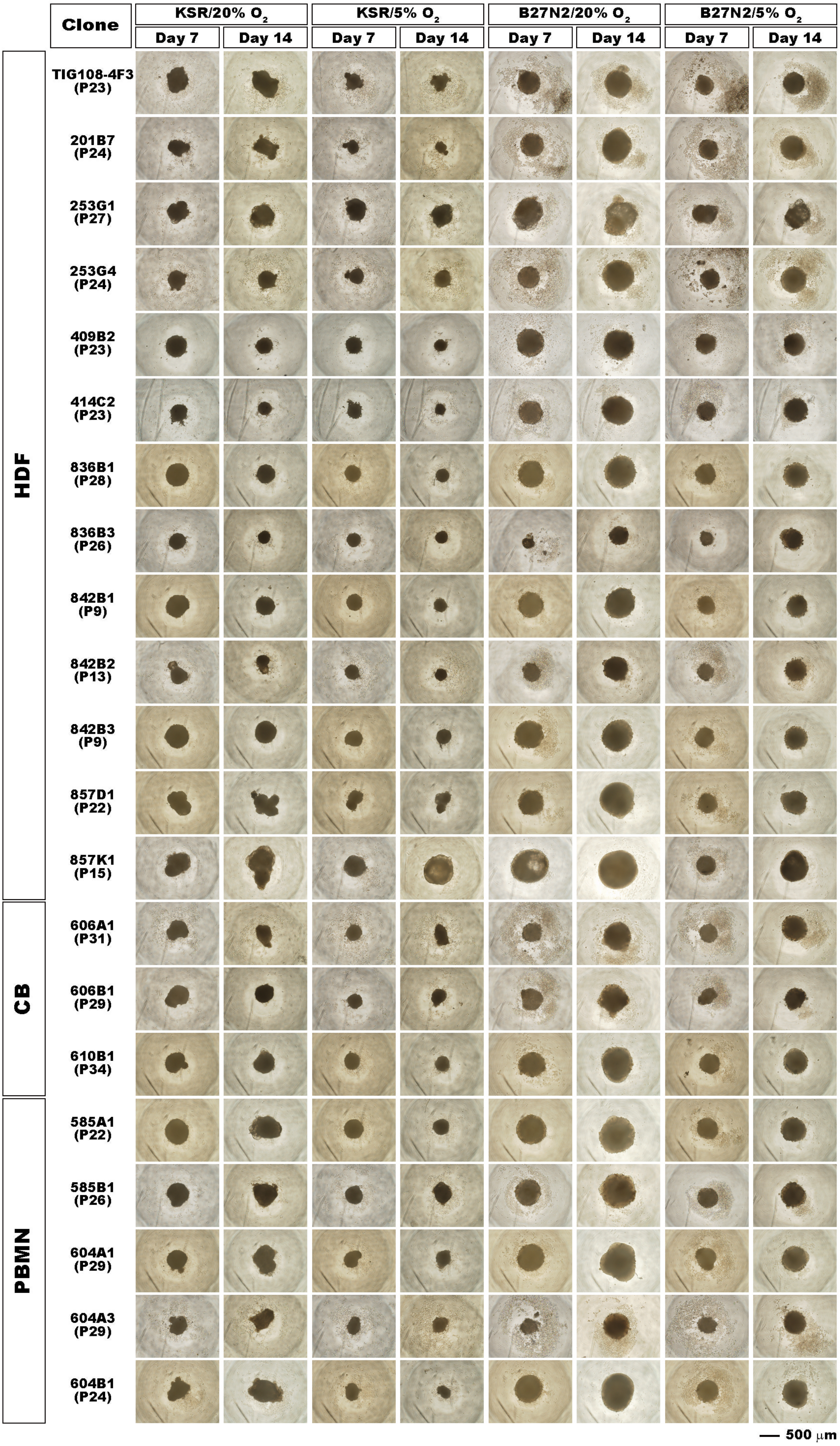
Clone
KSR/20% O2
KSR/5% O2
B27N2/20% O2
B27N2/5% O2
Day 7
Day 14
Day 7
Day 14
Day 7
Day 14
Day 7
Day 14
HDF
TIG108-4F3 (P23)
201B7 (P24)
253G1 (P27)
253G4 (P24)
409B2 (P23)
414C2 (P23)
836B1 (P28)
836B3 (P26)
842B1 (P9)
842B2 (P13)
842B3 (P9)
857D1 (P22)
857K1 (P15)
CB
606A1 (P31)
606B1 (P29)
610B1 (P34)
PBMN
585A1 (P22)
585B1 (P26)
604A1 (P29)
604A3 (P29)
604B1 (P24)
500 μm

Table S1, Related to Methods. Information of hiPSCs used in this study.

Clone	Passage no.	Vector	Factors	Feeder	Origin[a]	Age	Sex	Race[b]
TIG108-4F3	23	retrovirus	*Oct4, SOX2, KLF4, (M)*	SNL	HDF (TIG108)	40	F	J
201B7	24	retrovirus	*Oct4, SOX2, KLF4, c-Myc*	SNL	HDF (HDF1388)	36	F	C
253G1	27	retrovirus	*Oct4, SOX2, KLF4*	SNL	HDF (HDF1388)	36	F	C
253G4	24	retrovirus	*Oct4, SOX2, KLF4*	SNL	HDF (HDF1388)	36	F	C
409B2	23	plasmid	*Oct4, SOX2, KLF4, MYCL, LIN28, p53-shRNA*	SNL	HDF (HDF1388)	36	F	C
414C2	23	plasmid	*Oct4, SOX2, KLF4, MYCL, LIN28, p53-shRNA*	SNL	HDF (HDF1388)	36	F	C
836B1	28	plasmid	*Oct4, SOX2, KLF4, MYCL, LIN28, GLIS1*	SNL	HDF (HDF1388)	36	F	C
836B3	26	plasmid	*Oct4, SOX2, KLF4, MYCL, LIN28, GLIS1*	SNL	HDF (HDF1388)	36	F	C
842B1	9	plasmid	*Oct4, SOX2, KLF4, MYCL, LIN28, GLIS1*	SNL	HDF (TIG114)	36	M	J
842B2	13	plasmid	*Oct4, SOX2, KLF4, MYCL, LIN28, GLIS1*	SNL	HDF (TIG114)	36	M	J
842B3	9	plasmid	*Oct4, SOX2, KLF4, MYCL, LIN28, GLIS1*	SNL	HDF (TIG114)	36	M	J
857D1	22	plasmid	*Oct4, SOX2, KLF4, MYCL, LIN28, GLIS1*	SNL	HDF (TIG120)	6	F	J
857K1	15	plasmid	*Oct4, SOX2, KLF4, MYCL, LIN28, GLIS1*	SNL	HDF (TIG120)	6	F	J
606A1	31	plasmid	*Oct4, SOX2, KLF4, MYCL, LIN28, p53-shRNA*	SNL	CB CD34+ (donor a)	0	F	UN
606B1	29	plasmid	*Oct4, SOX2, KLF4, MYCL, LIN28, p53-shRNA*	SNL	CB CD34+ (donor a)	0	F	UN
610B1	34	plasmid	*Oct4, SOX2, KLF4, MYCL, LIN28, p53-shRNA*	SNL	CB CD34+ (donor b)	0	M	UN
585A1	22	plasmid	*Oct4, SOX2, KLF4, MYCL, LIN28, p53-shRNA*	SNL	PBMN αβT (donor x)	30s	M	J
585B1	26	plasmid	*Oct4, SOX2, KLF4, MYCL, LIN28, p53-shRNA*	SNL	PBMN αβT (donor x)	30s	M	J
604A1	29	plasmid	*Oct4, SOX2, KLF4, MYCL, LIN28, p53-shRNA*	SNL	PBMN αβT (donor y)	30s	M	J
604A3	29	plasmid	*Oct4, SOX2, KLF4, MYCL, LIN28, p53-shRNA*	SNL	PBMN αβT (donor y)	30s	M	J
604B1	24	plasmid	*Oct4, SOX2, KLF4, MYCL, LIN28, p53-shRNA*	SNL	PBMN αβT (donor y)	30s	M	J

a: HDF, Human Dermal Fibroblast; CB, Cord Blood; PBMN, Peripheral Blood MonoNuclear cell.

b: J, Japanese; C, Caucasian; UN, Unknown.

Table S2, Related to Figure 2. Comparison of two types of media and oxygen levels on the size of day 7 and day 14 neural aggregates. Data were analyzed with the Steel-Dwass test. The resulting p-values are shown for the statistically significant differences (*; $p < 0.05$, **; $p < 0.01$). Differences that were not statistically significant are indicated by dashes (-).

			KSR				B27N2			
			20% O_2		5% O_2		20% O_2		5% O_2	
			Day 7	Day 14	Day 7	Day 14	Day 7	Day 14	Day 7	Day 14
KSR	20% O_2	Day 7		-	1.35E-04**	4.26E-04**	-	6.49E-06**	-	2.49E-02*
		Day 14			4.99E-02*	3.16E-02*	-	3.10E-05**	-	-
	5% O_2	Day 7				-	1.86E-05**	8.10E-07**	9.15E-04**	8.10E-07**
		Day 14					1.51E-04**	5.68E-06**	3.47E-03**	1.43E-05**
B27N2	20% O_2	Day 7						2.15E-04**	8.89E-03**	-
		Day 14							8.10E-07**	8.48E-06**
	5% O_2	Day 7								2.73E-05**
		Day 14								

Table S3, Related to Figure 3. The neural aggregates were clustered based on *FOXG1* and *SOX1* expression levels using the K-medoids clustering method. Clones selected for further expansion are checked in the column on the right.

Cell	Condition	Origin	*FOXG1* : *SOX1*	Selected clones for further expansion
TIG108_4F3	BN/20%O2	HDF	High : High	-
TIG108_4F3	BN/5%O2	HDF	High : High	-
201B7	BN/20%O2	HDF	High : High	-
253G1	BN/20%O2	HDF	High : High	✓
409B2	KSR/20%O2	HDF	High : High	-
409B2	KSR/5%O2	HDF	High : High	✓
409B2	BN/20%O2	HDF	High : High	-
409B2	BN/5%O2	HDF	High : High	-
414C2	BN/20%O2	HDF	High : High	-
836B1	KSR/20%O2	HDF	High : High	-
836B1	KSR/5%O2	HDF	High : High	-
836B1	BN/20%O2	HDF	High : High	-
836B3	KSR/20%O2	HDF	High : High	-
836B3	BN/20%O2	HDF	High : High	-
836B3	BN/5%O2	HDF	High : High	-
842B1	KSR/20%O2	HDF	High : High	-
842B1	KSR/5%O2	HDF	High : High	-
842B1	BN/20%O2	HDF	High : High	-
842B1	BN/5%O2	HDF	High : High	-
842B2	KSR/20%O2	HDF	High : High	-
842B2	KSR/5%O2	HDF	High : High	-
842B2	BN/20%O2	HDF	High : High	-
842B2	BN/5%O2	HDF	High : High	-
842B3	KSR/20%O2	HDF	High : High	-
842B3	KSR/5%O2	HDF	High : High	-
842B3	BN/20%O2	HDF	High : High	-
606A1	KSR/20%O2	CB	High : High	✓
606A1	BN/20%O2	CB	High : High	-
606B1	BN/20%O2	CB	High : High	-
610B1	KSR/20%O2	CB	High : High	-
610B1	KSR/5%O2	CB	High : High	-
610B1	BN/20%O2	CB	High : High	-
610B1	BN/5%O2	CB	High : High	-
604A3	BN/20%O2	PBMN	High : High	✓
TIG108_4F3	KSR/5%O2	HDF	Middle : High	-
201B7	KSR/5%O2	HDF	Middle : High	✓
201B7	BN/5%O2	HDF	Middle : High	-
253G1	KSR/5%O2	HDF	Middle : High	-
253G1	BN/5%O2	HDF	Middle : High	✓
414C2	KSR/20%O2	HDF	Middle : High	-
414C2	KSR/5%O2	HDF	Middle : High	-
836B3	KSR/5%O2	HDF	Middle : High	-
842B3	BN/5%O2	HDF	Middle : High	-
857D1	KSR/5%O2	HDF	Middle : High	-
606A1	KSR/5%O2	CB	Middle : High	-
606A1	BN/5%O2	CB	Middle : High	✓
606B1	KSR/20%O2	CB	Middle : High	✓
606B1	BN/5%O2	CB	Middle : High	-
585A1	KSR/5%O2	PBMN	Middle : High	✓
585B1	BN/5%O2	PBMN	Middle : High	-
604A3	BN/5%O2	PBMN	Middle : High	✓
604B1	KSR/5%O2	PBMN	Middle : High	-
201B7	KSR/20%O2	HDF	Low : High	-
253G1	KSR/20%O2	HDF	Low : High	✓
253G4	KSR/20%O2	HDF	Low : High	-
253G4	KSR/5%O2	HDF	Low : High	-
253G4	BN/20%O2	HDF	Low : High	-
253G4	BN/5%O2	HDF	Low : High	✓

414C2	BN/5%O2	HDF	Low : High	-
836B1	BN/5%O2	HDF	Low : High	-
857D1	KSR/20%O2	HDF	Low : High	-
857D1	BN/20%O2	HDF	Low : High	-
857D1	BN/5%O2	HDF	Low : High	-
606B1	KSR/5%O2	CB	Low : High	✓
585A1	KSR/20%O2	PBMN	Low : High	-
585A1	BN/20%O2	PBMN	Low : High	-
585A1	BN/5%O2	PBMN	Low : High	-
585B1	KSR/20%O2	PBMN	Low : High	-
585B1	KSR/5%O2	PBMN	Low : High	-
585B1	BN/20%O2	PBMN	Low : High	-
604A1	KSR/20%O2	PBMN	Low : High	-
604A1	KSR/5%O2	PBMN	Low : High	-
604A1	BN/20%O2	PBMN	Low : High	-
604A1	BN/5%O2	PBMN	Low : High	✓
604A3	KSR/20%O2	PBMN	Low : High	-
604A3	KSR/5%O2	PBMN	Low : High	✓
604B1	KSR/20%O2	PBMN	Low : High	-
604B1	BN/20%O2	PBMN	Low : High	-
604B1	BN/5%O2	PBMN	Low : High	-
TIG108_4F3	KSR/20%O2	HDF	Low : Low	-
857K1	KSR/20%O2	HDF	Low : Low	-
857K1	KSR/5%O2	HDF	Low : Low	-
857K1	BN/20%O2	HDF	Low : Low	-
857K1	BN/5%O2	HDF	Low : Low	-

Table S4, Related to Experimental Procedures. Primers for quantitative RT-PCR.

Gene Symbol	Forward Primer (5' to 3')	Reverse Primer (5' to 3')
Oct4	GACAGGGGGAGGGGAGGAGCTAGG	CTTCCCTCCAACCAGTTGCCCCAAAC
NANOG	GCAGAAGGCCTCAGCACCTA	GGTTCCCAGTCGGGTTCAC
LIN28A	CACGGTGCGGGCATCTG	CCTTCCATGTGCAGCTTACTC
SOX17	CCCATAGTTGGATTGTCAAAACC	CACACCCAGGACAACATTTCTTT
T	TGGAATGCCTGCCCATC	CCGTTGCTCACAGACCACA
SOX1	AGCAGTTGTTTCTGGAAGAGTCTGT	AGGCCCTTATCCCGGACTAA
SOX2	ATGCACCGCTACGACGTGA	CTTTTGCACCCCTCCCATTT
PAX6	ACCTGGCTAGCGAAAAGCAA	CCCGTTCAACATCCTTAGTTTATCA
NES	CCAAGACTGCCCTGGAAAC	CCTCCCTCTCCAAGGAAACA
PROM1	GCACTTACGGCACTCTTCACC	CTATTCCACAAGCAGCAAAATCC
CDH2	GGACAGTTCCTGAGGGATCA	GGATTGCCTTCCATGTCTGT
BLBP	GGACTCTCAGCACATTCAAGAA	CCACATCACCAAAAGTAAGGGT
GFAP	ACATCGAGATCGCCACCTAC	ACATCACATCCTTGTGCTCC
S100B	CTCATCGACGTTTTCCACCA	CACAACCTCCTGCTCTTTGATTT
TUBA1A	AGCTCGGCAGTCGCGAAGCAG	CAATGACTGTGGGTTCCAAGTCTAC
TUBB3	GGGCCTTTGGACATCTCTTC	ACTCCTTCCGCACCACATC
DLX2	ACGCTCCCTATGGAACCAGTT	TCCGAATTTCAGGCTCAAGGT
FOXG1	CAAGGAGGGCGAGAAGAAGA	GATGAACTCGTAGATGCCGTTG
OTX1	GTGGCGCTCAAGATCAACC	TCCCGCACTGGAGAGGACTT
EN1	TGGGTGTACTGCACACGTTATTC	TGTCCTCCTTCTCGTTCTTCTTCT
GBX2	CCAAGTGGAAACGGGTGAA	ATGCTGACTTCTGATAGCGAACC
HOXC6	CCCAGGACCAGAAAGCCAGT	GGTCTGGTACCGCGAGTAGAT
GAPDH	CCACTTTGTCAAGCTCATTTCCT	TCTCTTCCTCTTGTGCTCTTGCT
